# The budding yeast *Saccharomyces cerevisiae* as a model organism: possible implications for gerontological studies

**DOI:** 10.1007/s10522-017-9712-x

**Published:** 2017-06-01

**Authors:** Tomasz Bilinski, Aneta Bylak, Renata Zadrag-Tecza

**Affiliations:** 1 Department of Biochemistry and Cell Biology, University of Rzeszow, Zelwerowicza 4, 35-601 Rzeszow, Poland; 2 Department of Ecology and Environmental Biology, University of Rzeszow, Zelwerowicza 4, 35-601 Rzeszow, Poland

**Keywords:** Aging, Alternative gerontology, Longevity, Senescence, Yeast

## Abstract

Experimental gerontology is based on the fundamental assumption that the aging process has a universal character and that the mechanisms of aging are well-conserved among living things. The consequence of this assumption is the use of various organisms, including unicellular yeast *Saccharomyces cerevisiae*, as models in gerontology, and direct extrapolation of the conclusions drawn from the studies carried on these organisms to human beings. However, numerous arguments suggest that aging is not universal and its mechanisms are not conserved in a wide range of species. Instead, senescence can be treated as a side effect of the evolution of specific features for systematic group, unrelated to the passage of time. Hence, depending on the properties of the group, the senescence and proximal causes of death could have a diverse nature. We postulate that the selection of a model organism to explain the mechanism of human aging and human longevity should be preceded by the analysis of its potential to extrapolate the results to a wide group of organisms. Considering that gerontology is a human-oriented discipline and that aging involves complex, systemic changes affecting the entire organism, the object of experimental studies should be animals which are closest relatives of human beings in evolutionary terms, rather than lower organisms, which do not have sufficient complexity in terms of tissues and organ structures.

## Introduction

Gerontology is a strongly human-oriented discipline that for many years has been attempting to find answers to several important questions, especially from the human being’s point of view, such as “Why do we age?” or “How long can we live?” Despite the sizeable body of knowledge collected over that time, these questions remain unanswered. The search for answers to these questions is far from simple because a human being cannot serve as an object of studies. This complication is the reason why, at the end of 1970s, a group of scientists assumed that, by analogy to other sub-disciplines of biological sciences, mechanisms of aging might be revealed by using simple model organisms in gerontological studies. This assumption, however, is rational only if mechanisms of aging had a universal character, i.e., were identical or at least very similar in both the model organism and humans, considering that gerontology is a human-oriented discipline. Numerous authors postulated ad hoc that aging is ubiquitous among animals and even fungi, including the unicellular yeast *Saccharomyces cerevisiae*. Consequently, they assumed that mechanisms of aging are well-conserved among living things and that conclusions drawn from studies carried out on the budding yeast may be extrapolated to human beings (Ganley et al. 2012; Kaeberlein 2012; Teplyuk 2012). It was also assumed that all adverse effects of performance of various life processes by yeast can be attributed to aging. One of such adverse effects of the *S. cerevisiae* cell’s performing its basic function—the mitotic reproduction—is reaching the cell reproduction limit, the value of which will depend on the genetic background of the cell. The approaching of the reproduction limit by each cell through completion of consecutive mitotic cycles was ultimately termed “replicative aging”. Such a name clearly suggests that the phenomenon must be causally connected to the aging process. Moreover, it was postulated that the reason why each cell of the budding yeast ceases reproducing after a number of mitotic cycles is the accumulation of a specific substance called “senescence factor” within the mother cell (Egilmez and Jazwinski 1989). Identification of such a “senescence factor” was one of the goals of gerontological studies on yeast (Aguilaniu et al. 2003; Erjavec et al. 2007; McFaline-Figueroa et al. 2011; Sinclair and Guarente 1997). It was assumed that revealing the nature of the hypothetical “senescence factor” would lead to the understanding of the nature of universal factors responsible for aging of all animals and fungi. Thus, during the ensuing years, gerontological studies focused on isolating mutant cells that produced more daughters than the standard strain cells (the so called “longevity” mutants) and establishing factors that determine longevity of the species (Kaeberlein 2010; Kaeberlein and Kennedy 2005; Kaeberlein et al. 2005; McCormick et al. 2015). These studies, however, were based on the assumption that the age of an individual and “longevity” of the population should be expressed as the number of daughters produced by a single cell, rather than the length of life, as in the case of animals. It seems that the need to confirm the existence of a hypothetical “senescence factor” as the causative agent of the “replicative aging” phenomenon served as the main rationale for extrapolating the scientific conclusions drawn from the obtained data to other living organisms. Some of the researchers were aware of the need to confirm the causal relation between the number of daughter cells produced and the longevity of the strain. The survey paper of the problem of the existence of the “senescence factor” as a causative agent of the phenomenon known as “replicative aging” that appeared in 2008 ends with a very symptomatic conclusion: “*With the advent of new technologies to isolate replicatively old budding yeast cells, progress in identifying senescence factors, age associated changes, and the mechanisms maintaining their asymmetry will accelerate*” (Henderson and Gottschling 2008). However, the expected methodological progress did not lead to unambiguous conclusions. On the contrary, results of new studies failed to confirm the causative role of some of the postulated “senescence factor” candidates (Ganley and Kobayashi 2014; Molon and Zadrag-Tecza 2016; Zadrag-Tecza and Skoneczna 2016). Another important element of the paradigm of yeast replicative aging was the postulate of existence of a specific mechanism responsible for transferring these factors to the mother cell or preventing their transfer to the daughter cell; the fact that the daughter cell was free of such factors ensured cell rejuvenation (Shcheprova et al. 2008; Zhou et al. 2011). Analysis of the data published over recent years shows that these two fundamental assumptions of yeast “replicative aging” need to be critically verified. First and foremost, the majority of the postulated candidates to the role of the “senescence factor” do not meet the initial basic criteria of “replicative aging” studies; furthermore, the mere existence of a specific mechanism responsible for asymmetric distribution of the postulated “senescence factors” between the daughter and the mother cell is highly controversial.

In this article, we present the arguments that form the basis for discussion about the limitations of the use of lower organisms as models for the studies of the mechanisms of aging. This is of particular importance in relation to the use organisms that are evolutionary distant from humans and having a different type of organism complexity or exhibiting different life strategies. We concentrate mainly on the phenomenon of longevity and the limitations of the use of unicellular organisms such as yeast *S. cerevisiae* in explanation of the mechanism of this process in human beings.

### Reproductive potential versus replicative aging

Most of previous studies concerning explanation of mechanisms of the reproduction limit in budding yeast cells pointed to the causal role of aging in the phenomenon referred to as “replicative aging”. However, the hypertrophy hypothesis casts doubt on the causal role of aging in this phenomenon, and suggests that the existence of the mitotic reproduction limit in the budding yeast cell is a consequence of the choice of budding as the mechanism of cytokinesis. Analysis of the budding process clearly shows that, in contrast to cells reproducing mitotically by binary fission, no mechanism of cell size reduction exists in the mitotic cycle of budding yeast cells. In other words, no cell division takes place during budding. Cell division means splitting the adult cell into two progenitor cells of identical size (symmetric cell division); in some cases, progenitor cells differ in size (asymmetric cell division). In budding yeast, the mother cell forms its daughter outside cell boundaries. The mitotic cycle ends by closing a very narrow channel known as the bud neck, or isthmus, through which the mother supplies the daughter during the S, G2 and partly the M phase of the cell cycle (Hartwell and Unger 1977; Johnston et al. 1977). The mitotic cycle of the budding yeast is frequently referred to as “asymmetric cell division”, wherein a programmable smaller daughter separates from the mother cell by sealing the bud neck of a fixed diameter, pre-existing since the S phase. Consequently, cell size is not reduced during the M phase. Naturally, both products of cytokinesis are not equal in size, but this “asymmetry” is not a consequence of the frequently postulated “asymmetric cell division”. The programmable smaller size of the daughter cell is a mechanism that prevents continuous increase of cell volume in the budding yeast population. Because of the absence of an emerging bud during the G1 phase of the mitotic cycle, the cell mass increase forces an enlargement of the mother cell, preferably during the same phase. After a number of cycles, each cell moves into the hypertrophy state which precludes further reproduction (Zadrag-Tecza et al. 2009). Consequently, highly oversized cells, which have performed a high number of cell cycles, are not able to reproduce efficiently. The hypertrophy hypothesis (Bilinski and Bartosz 2006; Biliński et al. 2012), which has an important experimental support (Yang et al. 2011; Zadrag-Tecza et al. 2009), clearly suggests that rather than aging, it is the choice of budding as an atypical mechanism of cytokinesis that causes limited reproduction capacity. For this reason, hypertrophy cannot be treated as a consequence of aging as was suggested by some of the yeast gerontologist (Ganley et al. 2012; Kaeberlein 2012). Hence, the studies on “replicative aging” of the budding yeast cannot answer most questions concerning the mechanisms of human aging and longevity.

### Yeast and human longevity

**Fig. 1:**
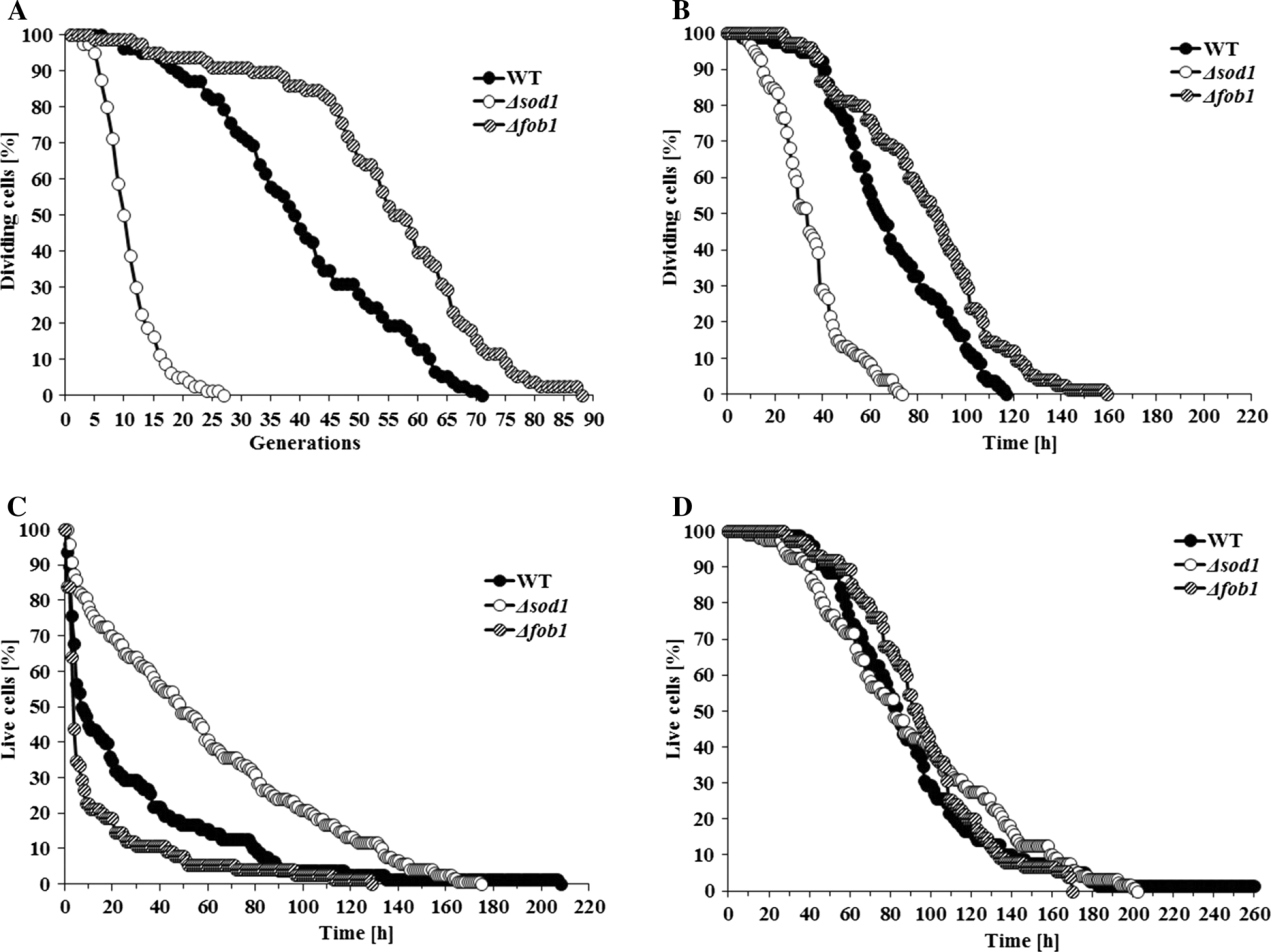
A comparison of two approaches for assessment the yeast lifespan of short-lived (∆*sod1* mutant), long-lived (∆*fob1*) and standard (SP4) strains: **a** reproductive potential expressed in the number of daughters, **b** reproductive phase of life, **c** post-reproductive phase of life, **d** total lifespan expressed in the units of time. Reproduced from: Zadrag-Tecza R, Molon M, Mamczur J, Bilinski T. “Dependence of the yeast *Saccharomyces cerevisiae* post-reproductive lifespan on the reproductive potential” published in Acta Biochimica Polonica  2013; 60(1):111–115, with permission

In yeast, “longevity” has been defined as the ability to produce more daughters compared to the standard strain. However, doubts concerning the use of the number of daughter cells as a measure of age or longevity were presented very early on, suggesting that it represents a measure of fecundity rather than age or longevity (Gershon and Gershon 2000, 2001). Using this unit of age, a number of genes were identified whose deletion resulted in increasing the number of daughters produced, e.g. *FOB1*, whose deletion leads to the reduction of rDNA recombination and consequently reducing the ERCs level (one of the postulated “senescence factor”) (Defossez et al. 1999); genes related to detection and metabolism of nutrients, such as *GPA2*, *GPR1*, *HXK2*, *SCH9*, or to regulation of cell growth in response to nutrient availability, such as *TOR1* (Kaeberlein and Kennedy 2005; Kaeberlein et al. 2005). Moreover, extensive studies conducted in 2015 demonstrated that the deletion of 238 genes resulted in an increase in replicative lifespan. The deleted genes were mainly ribosomal protein encoding genes or genes related to proteasomal degradation or mitochondria functions (McCormick et al. 2015). In turn, the analysis of yeast strains devoid of selected “longevity genes” showed that such strains live no longer than the standard strains of the same background when their length of life is expressed in the most natural units of age and longevity—that is, the units of time (Molon et al. 2015; Zadrag-Tecza et al. 2013). One can therefore conclude that the methodology used for measuring the lifespan of yeast does not represent the true length of a yeast cell’s life. The number of daughters produced by a single cell was termed “cell replicative lifespan” (RLS), and the phenomenon of approaching the limit of the replicative lifespan during each mitotic cycle was named “replicative aging”. The moment a cell reached the replicative limit was considered to be equivalent to the moment of the cell’s death. Several years later this assumption of yeast gerontology was questioned because sometimes there is a considerable time lapse between the moment the cell reaches the limit of its reproductive potential and the actual death of the cell (Minois et al. 2005). Consequently, lifespan of each individual cell of *S. cerevisiae* consists of reproductive and post-reproductive periods of life. The sum of the reproductive lifespan (RPLS) and post-reproductive lifespan (PRLS) is the total lifespan (TLS) expressed in units of time, equivalent to the term “lifespan” applied to animals (Zadrag et al. 2008). Studies of the length of these periods of life in various yeast strains, including studies of the known “longevity mutants” (those producing more daughters than the standard strain cells) have shown that the length of the post-reproductive lifespan is negatively correlated to the number of daughters produced (“replicative lifespan” or RLS), adopted as the measure of cell age and longevity of the strain according to the binding paradigm of yeast replicative aging studies. Consequently, the more daughters a cell of a given strain produces, the shorter it lives after ceasing to reproduce. An extensive analysis of lifespans of various strains differing in genetic backgrounds and bearing mutations changing the value of RLS (longevity-increasing genes or longevity-decreasing genes resulting in short-lived phenotypes) revealed that the value of the total lifespan expressed in units of time (hours) remains relatively constant). The comparison of values of replicative lifespan RLS (expressed in the number of daughters produced) and values of TLS (expressed in units of time) (Fig. 1) clearly demonstrates that the studied “longevity mutants” do not live longer than even the “short lived” mutants, when longevity is expressed in universal units of age, namely the units of time (Molon et al. 2015; Zadrag-Tecza et al. 2013).

Conclusions drawn from the studies of the mechanisms of thus defined longevity were extrapolated to animals under the assumption that the identified genes were associated with the universally understood “longevity”. A commonly used argument justifying such extrapolation has been the fact that some of these yeast “longevity genes” have homologues in other organisms, which opens a window for drawing general conclusions. The homology of genes between yeast and humans or the similarity of intracellular biochemical pathways is unquestionable, but their effects on a cell of a unicellular organism and a cell forming a larger body may be completely different because of the complications resulting from multicellularity and environmental conditions (Bilinski and Zadrag-Tecza 2014). Moreover, the longevity of humans does not measurably depend on the reproductive capacity of somatic cells. While human somatic cells show a limited reproductive potential similar to yeast, the consequences of this limit are completely different. Cellular senescence, the result of which is a terminal inability to perform cell division, is dependent among other factors on the shortening of telomeres caused by lack of active telomerase in most of the dividing cells (except for generative cells). In contrast, yeast cells have constantly active telomerase (Cohn and Blackburn 1995), which, however, does not protect them against the loss of reproductive potential. Furthermore, in a multicellular organism, senescent cells after terminal cell cycle arrest acquire of senescence-associated secretory phenotype traits (SASP). This creates grounds for numerous dysfunctions and diseases posing a real threat to the organism. In contrast, the same period of life of yeast cells is not taken into consideration due to the assumption that after the cell produces its last bud it may be considered dead. It is also worth emphasizing that studies based on model organisms such as yeast and widely accepted interpretation of replicative lifespan results show that the maximum lifespan is flexible, whereas the recent studies strongly suggest that the maximum lifespan of humans is fixed and has a natural limit (Dong et al. 2016).

The obvious inconsistencies in the interpretation of the results obtained in experiments based on the use of completely different units of age and longevity were also due to the extrapolation of the assumption that the existence of a cell reproduction limit (incorrectly recognised as cell death) must be a consequence of aging. However, such interpretation does not take into account that the number of generations may also depend on factors not directly related to the aging process. The hypertrophy hypothesis (Bilinski and Bartosz 2006; Biliński et al. 2012), since confirmed by an independent group of scientists (Wright et al. 2013; Yang et al. 2011), proposes an alternative explanation of the “replicative aging” phenomenon. According to that hypothesis, the existence of the limit of mitotic cycles that the cell can complete results from the cell reaching an enormous size precluding further reproduction. The cell size overgrowth after performing a number of mitotic cycles is a consequence of the choice of budding as the mechanism of cytokinesis, in which no cell volume reduction mechanism exists; instead, the cell has to increase its size during each G1 phase of the mitotic cycle. Consequently, the existence of the reproduction limit in each budding yeast cell is not causally related to the phenomenon of aging. Thus, the term “replicative aging” falsely suggests the existence of a causal relationship between the reproductive potential of the cell and longevity of the population. Similar conclusion can be found also in relation to cellular senescence in vitro. Cell cycle arrest does not always equal senescence (Blagosklonny 2011). Instead, loss of cell proliferative potential appears to be one of the consequences of cellular overactivation, which can lead to cellular hypertrophy (Demidenko et al. 2009). This point of view is supported by recent data showing that also replicative aging of the *Schizosaccharomyces pombe* yeast is not a consequence of an aging process (Spivey et al. 2017).

### Replicative versus chronological aging

The use of the budding yeast as a model organism of gerontology was not limited to the “replicative aging” studies. “*There are two primary ways to query the lifespan of this organism. If one asks how many times a cell can divide, the answer will be its replicative life span (RLS). If, on the other hand, one asks how long a cell can stay alive without dividing, the answer will be its chronological life span (CLS)*” (Polymenis and Kennedy 2012). Evidently, one question is missing: how long does a single budding yeast cell, considered as an individual, live, if its lifespan is expressed in units of time, as in the case of all other organisms? The longevity of individual yeast cells is expressed mainly in the number of daughters produced, not in units of time. Hence, the genes identified as resulting in an increased number of daughter cells are termed “longevity genes”, and assumed to effect longevity in animals, whose longevity, however, is expressed exclusively in units of time. Clearly, such a direct comparison does not consider two important aspects: first, that these units of measure are not tantamount; second, that the number of daughters produced (i.e., reproductive potential of the yeast cells) can also be regulated by mechanisms not dependent on the aging process.

Admittedly, the possibility of expressing lifespan and longevity in units of time exists in yeast gerontology. However, this model of yeast aging, named “chronological aging”, refers to the level of population, in contrast to “replicative aging”, where individual cells are analysed. This model was intended to reflect aging of post-mitotic (non-dividing) human cells (Longo et al. 1996, 2012). The model is based on stationary cell culture. Significant for the interpretation of results in this case is the fact that in such cultures cells differentiate into non-quiescent and quiescent subpopulations. The quiescent state relates to cells remaining in the G0 phase of the cell cycle, which is characterized by division arrest while preserving vital activity and the potential of returning to the cycle. A similar situation is also observed with *Metazoa* cells. However, this apparent similarity has completely different causes. In the case of yeast cells, the entry into the G0 phase is due to the depletion of nutrients; for *Metazoa* cells, the cause is lack of growth factors with full availability of nutrients. This is an expression of a different strategies pursued by uni- and multicellular organisms. A unicellular organism cannot control or maintain at a constant level the parameters of their environment—it may only monitor and adapt to variable environmental conditions. In contrast, a multicellular organism can control their internal environment and provide stable conditions for all cells. Thus, extremely divergent reasons for entering cells into quiescent state and differences in organism complexity can result in a completely different answer. Clearly, unfavourable conditions for yeast cells can trigger a stress response which will allow some cells to maintain their ability to survive. Consequently, the effects on both gene expression and cell metabolism are not the same for the yeast and *Metazoa* cells. Furthermore, quiescent-induced changes differ not only between yeast cells and *Metazoa* cells, but also among specific cell types, such as lymphocytes, fibroblasts, stem cells, and tumour cells (Valcourt et al. 2012).

The two models of yeast aging, “replicative” and “chronological”, were to correspond to the two types of cells of *Metazoa*, dividing and non-dividing, and thereby to enhance the applicability of the results to human aging. In addition, it was suggested that a functional relationship may exist between these two types of yeast aging models (Polymenis and Kennedy 2012). Unfortunately, the available evidence for that is quite unconvincing, as these models refer to completely different levels of biological complexity—an individual and a population. In the case of studies of “replicative aging”, single cells are mortal while the population remains immortal. In contrast, in “chronological aging” studies, the whole population dies as a consequence of the growing cells depleting all nutrients and the resulting intoxication by acetic acid, a product of yeast metabolism. Death resulting from depletion of nutrients does not reflect the situation of post-mitotic cells of the human body, which are in the state of constant homeostasis maintained by precise control of nutrient concentration in the body fluids. In such case, increase in the yeast chronological lifespan should rather be explained in terms of increase of stress resistance than as slow-down of the aging process. The claim that such a form of death may result from aging processes as seen from the human perspective seems somewhat controversial. In other words, the conclusions drawn from the results of such studies are difficult to apply to human gerontological studies.

### Longevity regulation in animal world

**Fig. 2:**
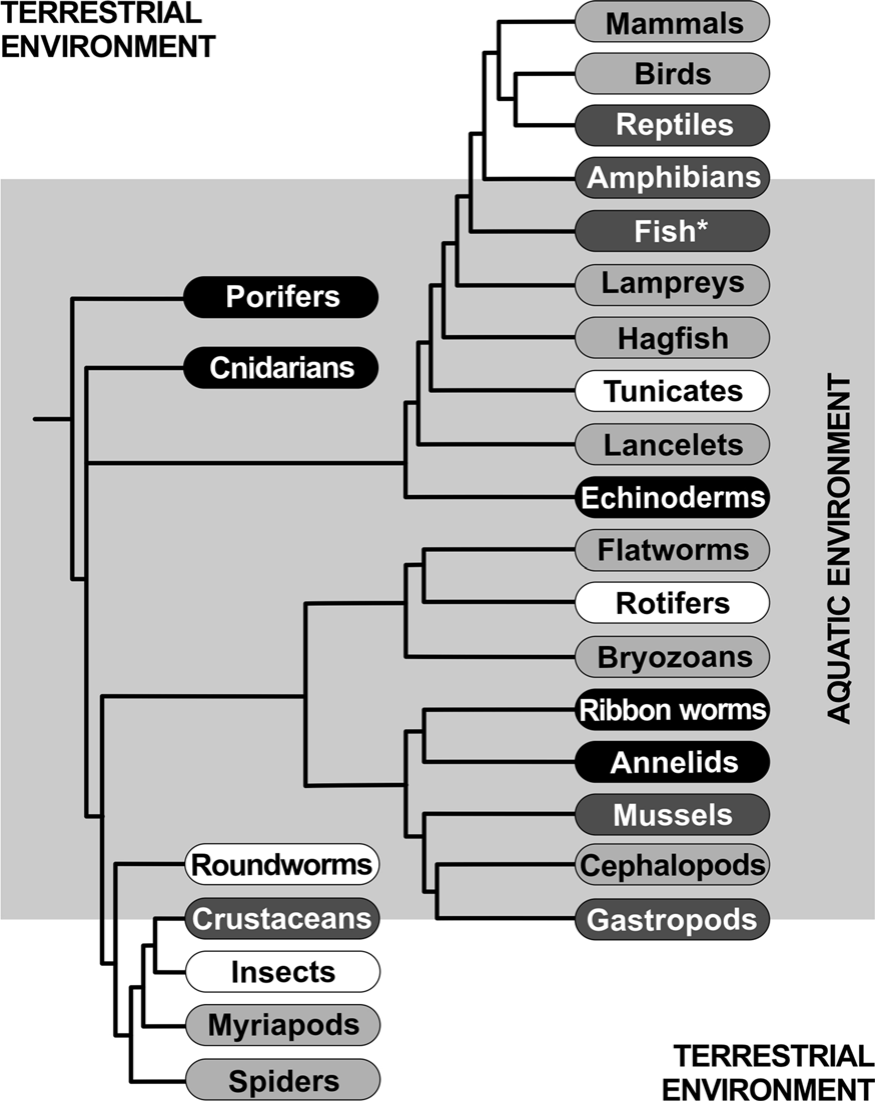
Lifespan of different groups of animals; labels: *black* biologically immortal, *dark grey* long lifespan, *light grey* medium lifespan, *white* short lifespan, *cartilaginous fish, lobe-finned fish, and ray-finned fish

The assumption of the universality of the aging process virtually allows for the use of biological objects so different in terms of chemical and structural complexity as the single-celled baker’s yeast as models in gerontological studies to explain the mechanism of human aging. The literature abounds in papers emphasising the enormous potential of studies based on budding yeast, and the conclusions, concerning among others “longevity genes”, are extrapolated directly to the longevity of animal organisms. The validity of this type extrapolation is based mainly on the assumption of the universality of aging mechanisms (Fig. 2). Therefore, one might ask whether the phenomenon of yeast “replicative aging” is causally associated with the phenomenon known as aging from the human perspective, as “universal” includes “human” (Bilinski and Zadrag-Tecza 2014). Analysis of life expectancy of organisms from different taxonomic groups and their life strategies determined by environmental conditions (Fig. 2) indicates quite clearly that the aging process is neither universal, nor its mechanisms are conserved (Bilinski et al. 2016).

**Fig. 3:**
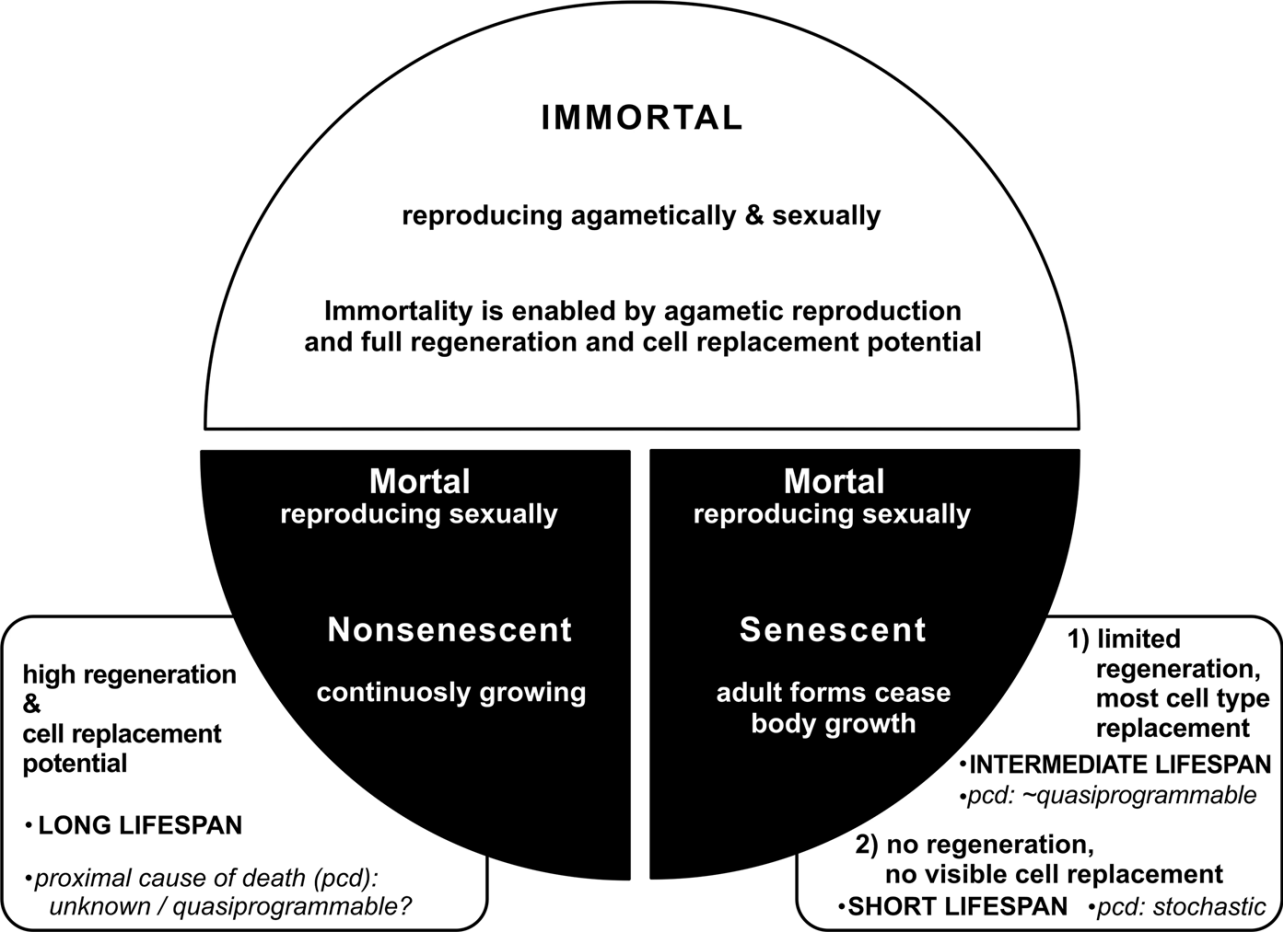
Classification of organisms due to the type of senescence and longevity

Longevity in the animal world is not uncommon, as demonstrated by numerous examples. However, these examples cannot be explained by a purely reductionist approach to the problem. First, it is necessary to analyse a higher level of nature organisation—the population. Only in this way—by explaining the genesis of selected phenotypes—may one effectively explain the phenomenon of longevity. In addition, the analysis of life expectancy of some species of animals should take into account both the conditions in which such species live and their development programs because without considering these two aspects it is impossible to identify key factors influencing their genesis (Fig. 3).

In animals, two basic strategies of development may be observed. The first such strategy, used mainly in most insects, precludes longevity of the adult forms (imago). This is due to the reconstruction program body in the larval stage, which results in the soma of the adult form composed only of post-mitotic cells. Obviously there are exceptions to this rule: in some long-lived forms, such as termite queens, high degree of body size increase is observed in imagoes, but it is a rather unique phenomenon among eusocial species of insects. The second strategy is observed in representatives of different taxonomic groups, whose life program allows for longevity, or even “biological immortality”, which means absence of quasi-programmable factors forcing death (intrinsic factors of death) (Bilinski et al. 2016). Examples of “biological immortality” are observed mainly in the lower invertebrates (Martinez 1998), while examples of longevity are found in most systematic groups, among others, echinoderms, crustaceans and vertebrates, namely fish, amphibians and reptiles. A common feature of all these animals is that they retain the ability to increase their body size throughout their lives, specifically, well after puberty (Bilinski et al. 2015, 2016). This feature may be similar to adolescence in humans and other mammals. Symptoms of aging can only be seen after reaching full maturity. The preceding period is a time during which both the maturing and growing organism sends the necessary signals, and the individual parts of the body are able to receive them, and therefore are capable of renewal. Unlike reptiles, mammals have kept only an elementary range of regeneration capacity. In contrast, crustaceans, fish, amphibians and reptiles, beside the ability to send and receive signals, have preserved a permanently active program of implementation of their body plan. In this way, even non-existent parts of the body can be restored, such as legs or even a tail. In this group of organisms, a phenomenon of “negligible senescence” may be observed, i.e., no visible symptoms of senescence at the organismal level (Finch et al. 1990). These symptoms observed at the cellular level (waste retention) may possibly be periodically removed as part of broader reconstruction carried out during growth.

These two postulated development strategies either determine the inability of developing the longevity phenotype in adult forms (insects), or only enable evolution of the phenotype in other forms. Creation of the adult form composed entirely of post-mitotic cells is therefore a sufficient condition for the evolutionary irreversible short-lived phenotype. On the other hand, the continuous growth ability program is only a sine qua non condition for achieving the longevity phenotype.

## Conclusion

While aging is undoubtedly a ubiquitous phenomenon in biology, it is not universal, and its mechanisms are not conserved in a wide range of species. Moreover, senescence is not a genuine trait, but rather a side effect of the evolution of specific features for systematic groups, unrelated to the passage of time. Thus, depending on the properties of the systematic group, the process known as senescence and the proximal causes of death have a diverse nature. Therefore, the selection of a model organism to explain the mechanism of aging and human longevity should be preceded by a broad analysis of its potential to extrapolate the results to a wide group of organisms. Crucial for that decision will be a consideration of whether the selected organism is sufficiently complex in terms of tissues and organ structures. The budding yeast exhibits some features which have been considered suitable for a model organism in aging-related research, including the possibility of a direct genotype to phenotype analysis, short generation time, small genome, possibilities of a wide range of genetic manipulations and large number of characterised mutants, and homology of sequence of a number of genes between yeast and humans, but these features do not constitute a counterweight to the undisputed limitations of their use. These limitations are particularly significant when considering these aspects of aging that arise from complex intercellular interactions in multicellular organisms. Above all, as a unicellular organism, yeast lack an intercellular inflammatory signalling system and senescence-associated secretory phenotype, which provides grounds for several dysfunctions and diseases constituting a real threat to the organism.

Because of these aspects, the budding yeast is a very popular model organism used in many branches of science as a model of the eukaryotic cell for analysis of the physiological, biochemical, genetic or molecular aspects; however, its usefulness in explaining the mechanisms of aging and longevity of multicellular organisms is very limited. Consequently, the object of experimental gerontology studies should be animals that are the closest relatives of human beings in evolutionary terms. An important aspect of biogerontological studies should be the search for factors that reduce the negative effects of aging, which might contribute to improving the quality of life and slowing down senescence, rather than prolongation of the maximum lifespan of the species.
